# Malaria an opportunistic infection in HIV/AIDS patients? – A Nigerian experience

**DOI:** 10.4102/ajlm.v11i1.1842

**Published:** 2022-11-24

**Authors:** Joseph N. Enuma, Felix O. Sanni, Malau B. Matur, Njab E. Jean, Tosan Erhabor, Iheukwumere I. Egbulefu

**Affiliations:** 1 Department of Biological Sciences, University of Abuja, Abuja, Nigeria; 2 Department of Research and Development, Fescosof Data Solution, Ota, Ogun State, Nigeria; 3 Department of Zoology, University of Jos, Jos, Plateau State, Nigeria; 4 Laboratory Department, Total Laboratory Project for Africa, Abudja, Nigeria; 5 Department of Haematology, Medical Laboratory Science Council of Nigeria, Abuja, Nigeria; 6 Laboratory Service, Labhub Medical & Diagnostic Centre-Nigeria, Abuja, Nigeria

**Keywords:** malaria, infection, HIV, opportunistic, CD4

## Abstract

**Background:**

HIV and malaria interact at the level of the host’s susceptibility to infection, but little is known about the effect of HIV on malaria infection in Nigeria.

**Objective:**

This study estimated the prevalence of malaria parasitaemia and its relationship with HIV immunodeficiency.

**Methods:**

This cross-sectional study was conducted in two hospitals in Abuja, Nigeria between October 2012 and March 2013 among 600 respondents, comprising 200 HIV-negative controls, 200 HIV-positive patients on antiretroviral therapy (ART), and 200 HIV-positive patients not on ART. Malaria parasites, malaria density and absolute CD4 counts were carried out on all three groups. Participants with CD4 counts below 350 cells/mm^3^ were considered immunocompromised and likely to develop opportunistic infections.

**Results:**

Most study participants were aged 21–40 years (65.2%). The mean CD4 counts of HIV-positive patients not on ART (300 ± 211 cells/mm^3^) and those on ART (354 cells/mm^3^) were significantly lower than among controls (834 cells/mm^3^) (*p* < 0.001). Malaria prevalence was not statistically different between the controls (44.5%), patients on ART (40.5%), and those not on ART (39.5%) (*p* = 0.562). Compared to 7% immunodeficiency among controls, 56% of patients on ART and 65.5% of those not on ART had a CD4 count < 350 cells/mm^3^ (*p* < 0.001). The prevalence of malaria parasitaemia among immunodeficient individuals (42.4%) was similar to prevalence among those with CD4 counts > 350 cells/mm^3^ (40.8%; *p* = 0.695).

**Conclusion:**

These findings suggest that malaria parasitaemia is not an opportunistic infection among HIV-positive individuals in Nigeria.

## Introduction

With about 14% of the world’s population, sub-Saharan Africa is home to roughly 67% of all people living with HIV/AIDS.^1,2^ On the other hand, the World Health Organization estimates that sub-Saharan Africa accounts for more than 90% of the worldwide burden of malaria.^3^ As a result, any interaction between the two infections is expected to have a considerable impact on health in the region, particularly in countries like Nigeria, which now has the largest malaria burden in sub-Saharan Africa and ranks first among African countries affected by HIV/AIDS.^3^

Opportunistic infections occur more frequently and with greater severity in individuals with compromised immune systems, such as those positive for HIV.^4^ Opportunistic infections are less prevalent today than during the early stages of HIV and AIDS, owing to improved medications that reduce HIV viral load and strengthen the immune system.^5^ However, many people living with HIV continue to develop opportunistic infections, because they are unaware of their HIV status, are not on treatment, or their treatment does not keep their viral load low enough for their immune system to fight infections.^6^

The possible repercussions and public health impact of HIV and malaria’s geographical overlap have been identified and explored in recent years.^5,7,8^ The two diseases interact at the level of the host’s susceptibility to infection. HIV infection increases the probability of clinical and severe malaria, while *Plasmodium falciparum* infection increases HIV viral load.^8,9^ Additionally, dual infection has been found to contribute to the spread of both illnesses in sub-Saharan Africa, raising concerns about treating coinfected patients and the possibility of therapeutic interactions.^10^ Because of the significant geographic and social overlap between people infected with HIV and malaria, complex interactions at the individual and group levels are possible.^11^ Notably, any relationship between the two infections has the potential to have considerable public health implications due to their respective frequencies.^12^

HIV infection may exacerbate malaria’s burden by increasing susceptibility to infection or impairing antimalarial medications’ preventative and therapeutic efficiency, as both are immune-mediated.^13^ However, early 1990s investigations failed to reveal substantial interactions between malaria and HIV in coinfected children and adults who had acquired semi-immunity to malaria, probably due to research design problems and a lack of information about immunosuppression.^14^ In the early 2000s, it was shown that HIV infection has a clinical effect on malaria infection and disease and that this effect appears to be reliant on the dynamics of malaria transmission and the degree of HIV-associated immunosuppression.^15,16^ HIV-positive individuals who have not acquired immunity to malaria exhibit a significant increase in the severity of infection, in contrast to those who have naturally acquired immunity to malaria, for whom HIV infection is associated with just a mild rise in clinical disease.^17^ Malaria has been identified as a risk factor for concurrent HIV infection at the population level in the recent past.^13^ Studies have addressed the prevalence of asymptomatic malaria,^18,19^ association between clinical malaria and immunosuppression among HIV-positive patients.^20^ and effects of malaria infection on HIV-positive individuals in Nigeria,^4,18,19,21,22^ but information on malaria as an opportunistic infection in HIV-positive individuals is scanty. As such, this study sought to estimate the prevalence of malaria parasitaemia and its relationship with HIV immunodeficiency among HIV-positive patients in Abuja, Nigeria.

## Methods

### Ethical considerations

This study received approval from the Federal Capital Territory Health Research Ethics Committee (FHREC) with approval number FHREC/2012/11/34/05–11-12 The study protocol was made clear to the participants, and their informed consent was obtained, both orally and in written forms, before recruiting them to the study. Information that could be traced to any of the respondents was not collected. Data obtained from the participants were treated confidentially and for research purposes only.

### Study design

This prospective, randomised, cross-sectional study assessed malaria as an opportunistic infection in HIV-positive patients in Abuja, Nigeria, between October 2012 and March 2013. Abuja is home to all of the country’s ethnic groups and six geographical regions. The study investigated two main groups: HIV-positive patients and HIV-negative individuals attending the HIV/AIDS clinics in either the Wuse General Hospital or Asokoro District Hospital. These hospitals are central to the Federal Capital Territory and represent Nigeria’s Abuja metropolis. A total of 600 individuals were recruited to the study by using a convenience method to select participants by asking consented respondents if they were on antiretroviral therapy (ART) or not until the required number of participants was completed, to ensure equal numbers of participants in each of three groups: HIV-negative, HIV-positive on ART, HIV-positive not on ART. HIV-negative individuals served as controls for the study. Only HIV-positive and HIV-negative patients attending the two hospitals were included in the study; those who did not attend these hospitals were excluded.

### Data collection

After consent was given and before being enrolled into the study, HIV tests were done for the control patients to determine that they were truly HIV-negative and for the HIV-positive patients not on ART to confirm they were truly HIV-positive. HIV tests were not conducted on HIV-positive patients on ART, because we confirmed their status from the treatment case files retrieved from the hospitals. All participants were tested for the presence and density of malaria parasites and for CD4 count to assess their immune status. Age and gender were collected from patients’ medical records.

### Experimental procedures

#### Collection of blood samples for HIV test

A 5 mL blood sample was collected into an ethylenediaminetetraacetic acid tube by the staff of the laboratory departments at both hospitals for all three tests: HIV test, malaria parasite examination and CD4 count. Samples that could not be tested immediately were stored in a refrigerator at 2 °C – 6 °C and tested within 48 h. Blood samples were tested at the same facility in which they were collected and were not transported to another facility.

#### HIV testing

Three test kits, namely, Determine HIV 1/2 (Abbott Japan Co., Ltd., Tokyo, Japan), Uni-Gold HIV 1/2 (Chembio Diagnostic Systems, Inc. Hauppauge, New York, United States) and Stat Pak HIV 1/2 (Trinity Biotech Plc., Bray, Co. Wicklow, Ireland), which are the National HIV Testing Algorithm Screening Tests^23^ (First-line test: Determine HIV 1/2; Confirmatory test: Uni-Gold HIV 1/2; Tie Breaker test: Stat Pak HIV 1/2) used for the HIV testing. Whole blood was used for the testing. Using the Determine kit, one drop of whole blood was put onto the sample compartment of the kit, and two drops of chase buffer were added and allowed to run for 10 min before the reading was taken. The appearance of two pink lines on the strip indicates that the sample is positive; only one line indicates the test is negative. If there is no line at all, the test is considered invalid. Positive results were confirmed with the confirmatory test kit (Uni-gold). Since we need HIV-negative participants in this group to serve as inherent controls, patients with HIV-positive results were excluded from the study.

#### Malaria parasite examination

One or two drops of venous blood were spread on a clean, dry slide to make a thick blood film. The film was allowed to air dry, and the slide was then fixed in 100% alcohol for approximately 30 min. The slide was then flooded with a fresh solution of 10% Giemsa stain (Merck KGaA, Darmstadt, Germany), freshly prepared in distilled water. The stain was allowed to remain for approximately 30 min. After 30 min, the slide was rinsed in tap water and allowed to air dry thoroughly. When the thick film was completely dried, a drop of oil immersion was applied to an area of the film which appeared mauve coloured (usually around the edges). The oil was spread to cover about 10 mm in diameter to allow the film to be examined first at low magnification (Olympus microscope, New York Microscope Company, Hicksville, New York, United States). The area that was well stained but not too thick was selected. A 100X objective lens was used to examine malaria parasites and malaria pigments. At least 100 high power microscope fields were examined for parasites. The approximate numbers of parasites (trophozoites, schizonts and gametocytes) and whether malaria pigment was present in the white cells were reported, with the result being graded as either + (mild or moderate parasitaemia), ++ (severe parasitaemia) or +++ (very severe parasitaemia). When no malaria parasites were seen, the sample was reported as ‘none seen’.^24^

#### Estimating malaria parasites numbers (density)

A drop of immersion oil was placed on the stained slide and viewed under a 100× objective lens. Two hundred (200) leucocytes were counted while noting the number of malaria parasites (trophozoites and schizonts) present during the count. The number of parasites per microlitre was calculated using the formula:


$$Nx\frac{T}{L}$$ [Eqn 1]

Where *N* = number of parasites counted; *T* = Assumed total white blood cell (leucocyte) count (usually 8000) in cells/µL, and *L* = Number of Leucocytes counted.^24^

#### Estimation of CD4

The flow cytometer (Sysmex Partec’s CyFlow™ Counter System, Sysmex, Kobe, Japan) was prepared for analysis, and the operating software was prepared for measurement. The sample tube was filled with prepared sample suspension (20 µL of CyFlow™ CD4 PE antibody plus 20 µL thoroughly mixed whole blood). The mixture was allowed to incubate in the dark for 15 min, mixing the sample every 5 min during incubation. Then 800 µL of CD4 buffer was added and mixed gently. The tube was filled not to more than 2/3 of its volume. The sample was inserted onto the sample port until a ‘click’ sound was heard. The sample was fully mounted within one second. The measurement (acquisition) started automatically; the operating software indicated the Prerun, Run and Count Status. In the Prerun phase, cells were quickly transported to the flow cuvette’s analysis position; they were analysed and classified into histograms on real-time display in the run phase and counted for a given volume (in a cubic millilitre of blood) in the final stage. After the count phase, the acquisition finished automatically. The button end was clicked to complete the acquisition manually, or the sample tube was removed from the sample port. After each day run, 1.6 mL of cleaning fluid and 1.6 mL of sheath fluid were used for cleaning.^25^

### Data analysis

Data were collected and cleaned in Microsoft Excel (Microsoft Corp., Redmond, Washington, United States) before they were exported to IBM-SPSS Statistics for Windows (Version 25.0., released 2017; IBM, Armonk, New York, United States) for analysis. In the analyses, HIV-positive participants were categorised as being on ART or not on ART according to their self-reported status at the time of recruitment. Participants were categorised as immunodeficient if they had a CD4 count of less than 350 cells/mm^3^ and were HIV-positive. The Chi-square test was used to compare malaria and immune suppression proportions in the study groups. Analysis of Variance was used to compare the mean CD4 counts in the study groups, using a 95% confidence level, and a *p*-value of less than 0.05 was considered significant.

## Results

Most study participants were aged 21–40 years (65.2%) (Table 1). This age group was predominant across all study groups, constituting 49.5% of controls, 75% of HIV-positive patients on ART and 71.0% of patients not on ART. Similarly, most study participants were female (69.0%), constituting 61.5% of controls, 74.0% of HIV-positive patients on ART and 71.5% of HIV-positive patients not on ART.

**TABLE 1:** Sociodemographic characteristics of controls, HIV**-**positive patients with and without antiretroviral therapy, Abuja, Nigeria in 2012–2013.

Characteristics	Controls		On ART†		Not on ART†		Total	
	*n*	%	*n*	%	*n*	%	*n*	%
**Age category**								
0–10 years	39	19.5	6	3.0	19	9.5	64	10.7
11–20 years	17	8.5	2	1.0	6	3.0	25	4.2
21–30 years	63	31.5	61	30.5	72	36.0	196	32.7
31–40 years	36	18.0	89	44.5	70	35.0	195	32.5
41–50 years	25	12.5	30	15.0	25	12.5	80	13.3
51–60 years	14	7.0	11	5.5	6	3.0	31	5.2
Above 60 years	6	3.0	1	0.5	2	1.0	9	1.5
**Gender**								
Female	123	61.5	148	74.0	143	71.5	414	69.0
Male	77	38.5	52	26.0	57	28.5	186	31.0

ART, antiretroviral therapy.

† , Self-reported ART status.

The mean CD4 counts of HIV patients not on ART (300 ± 211 cells/mm^3^, i.e., immunodeficient) and those on ART (mean: 354 cells/mm^3^) were significantly lower than the control group (mean: 834 cells/mm^3^) (*p* < 0.001) (Table 2). On the other hand, malaria prevalence was not significantly different across all study groups (controls, 44.5%; patients on ART, 40.5%; those not on ART, 39.5%; (*p* > 0.562). Compared to 7% immunodeficiency recorded among the control group, more than half of HIV-positive patients on ART (56.0%) and 65.5% of those not on ART were immunodeficient (*p* = 0.001).

**TABLE 2:** Prevalence of malaria and immunodeficiency status among controls and HIV-positive patients with and without antiretroviral therapy, Abuja, Nigeria in 2012–2013.

Parameters	Controls			On ART†			Not on ART†			Total				
	*n*	%	Mean ± s.d.	*n*	%	Mean ± s.d.	*n*	%	Mean ± s.d.	*n*	%	*p*-value	*F*-value	*χ*^2^-value
**CD4 count (cells/mm^3^)**	-	-	834 ± 378	-	-	354 ± 207	-	-	300 ± 211			< 0.001*	223.8	-
**Malaria parasite prevalence**														
Positive	89	44.5	-	81	40.5	-	79	39.5	-	249	41.5	0.562	-	1.15
Negative	111	55.5	-	119	59.5	-	121	60.5	-	351	58.5			
**Immunodeficiency status**														
Positive (CD4 < 350)	14	7.0	-	112	56.0	-	131	65.5	-	257	42.8	< 0.001*	-	161
Negative (CD4 ≥ 350)	186	93.0	-	88	44.0	-	69	34.5	-	343	57.2			

ART, antiretroviral therapy; s.d., standard deviation; *F*, analysis of variance (ANOVA); *p, p*-value; *χ*^2^, Chi-square value.

* , Significant at *p* < 0.001.

† , Self-reported ART status.

The prevalence of malaria parasitaemia among immunodeficient individuals was 42.4%, which was not significantly different (*p* = 0.695) from the 40.8% among those with a CD4 count of 350 cells/mm^3^ and above (Table 3).

**TABLE 3:** Prevalence of malaria parasitaemia across immunodeficiency status of all participants in Abuja, Nigeria in 2012–2013.

Variable	Immunodeficient†				Total		*χ* ^2^	*p*
	Positive		Negative					
	*n*	%	*n*	%	*n*	%
**Malaria parasite test**							0.150	0.695
Negative	148	57.6	203	59.2	351	58.5	-	-
Positive	109	42.4	140	40.8	249	41.5	-	-

*χ*^2^, Chi-square value.

† , Immunodeficiency was defined as a CD4 count of < 350 cells/mm^3^.

Most malaria cases across all groups were found within the group aged 21–40 years, including 52.8% of controls, 79.0% of HIV patients on ART, 75.9% of HIV-positive patients not on ART and 77.5% of all HIV-positive patients (*p* = 0.001) (Table 4). Similarly, the group aged 21–40 years also comprised the majority of immunodeficient participants (controls, 64.3%, HIV-positive patients on ART, 73.3%, HIV-positive patients not on ART, 74.8%, and all HIV-positive patients, 74.1%).

**TABLE 4:** Comparison of malaria and immunodeficiency with age of HIV-positive and HIV-negative individuals in Abuja, Nigeria 2012–2013.

Variable	Controls		On ART†		Not on ART†		HIV-positive		*p*
	*n*	%	*n*	%	*n*	%	*n*	%	
**Malaria parasite positive (Overall [*n* = 249, 41.5%])**									
Age category									0.001*
0–10 years	15	16.9	2	2.5	5	6.3	7	4.4	-
11–20 years	9	10.1	0	0.0	3	3.8	3	1.9	-
21–30 years	31	34.8	31	38.3	29	36.7	60	37.5	-
31–40 years	16	18.0	33	40.7	31	39.2	64	40.0	-
41–50 years	11	12.4	13	16.0	7	8.9	20	12.5	-
51–60 years	4	4.5	2	2.5	2	2.5	4	2.5	-
Above 60 years	3	3.4	0	0.0	2	2.5	2	1.3	-
**Immunodeficient‡ (Overall [*n* = 257, 42.8%])**									
Age category									0.435
0–10 years	1	7.1	0	0.0	3	2.3	3	1.2	-
11–20 years	1	7.1	1	0.9	2	1.5	3	1.2	-
21–30 years	4	28.6	35	31.3	48	36.6	83	34.2	-
31–40 years	5	35.7	47	42.0	50	38.2	97	39.9	-
41–50 years	3	21.4	20	17.9	23	17.6	43	17.7	-
51–60 years	0	0.0	8	7.1	4	3.1	12	4.9	-
Above 60 years	0	0.0	1	0.9	1	0.8	2	0.8	-

ART, antiretroviral therapy.

* , Significant at *p* < 0.001.

† , Self-reported ART status.

‡ , Immunodeficiency was defined as a CD4 count of < 350 cells/mm^3^.

## Discussion

The mean CD4 count among our study controls was 834 cells/mm^3^; it was 354 cells/mm^3^ among HIV-positive patients on ART, and 300 cells/mm^3^ among HIV-positive patients not on ART, which shows that those HIV-positive patients not on ART were immunodeficient. Several studies have supported this finding, reporting how a CD4 count of less than 350 cells/mm^3^ makes a patient immunodeficient, elevating their chances of being susceptible to different diseases.^20,26^ A study conducted in India reported that a lower CD4 count increases the chance of infection among HIV-positive children below the age of 14 years.^27^ They found the prevalence of opportunistic infections in 90.91% of the HIV-positive patients with a CD4 count of less than 200 cells/mm^3^, 21.21% among those with a CD4 count between 200 and 349 cells/mm^3^, and 23.81% among those with a CD4 count of 350 cells/mm^3^ – 499 cells/mm^3^, while patients with CD4 counts < 500 cells/mm^3^ had the least amount of infection (11.43%).^27^ This finding may also explain why HIV clients not on ART with a CD4 count of less than 350 cells/mm^3^ had a higher percentage of immunodeficiency.

However, with a lowered immunity (lower mean CD4 count) among HIV-positive patients on ART and those not on ART, one would ordinarily expect a higher prevalence of malaria parasitaemia in these groups, but this was not the case in our study. The study found that malaria prevalence was higher among controls and HIV-positive patients on ART, which might be explained by various factors, including the number of respondents in each group. Additionally, genotype may play a role, as it has previously been reported that individuals with AA genotype are more susceptible to malaria.^28^ This claim was further substantiated by stating that malaria parasites do not thrive in individuals with sickle cell trait, since haemoglobin ‘S’ is known to inhibit the growth and reproduction of malaria parasites, which may explain the finding of a higher percentage of controls have the AA genotype.^28^ ART may also potentially impact an individual’s vulnerability, as studies have shown that it is less effective against the asexual ring stages of *P. falciparum* in the blood.^29,30^

The prevalence of parasitaemia was not significantly higher among the group that was both HIV-positive and immunodeficient than in the negative group, which shows malaria is not an opportunistic infection in HIV-positive individuals. The study also found that most respondents who tested positive for malaria in all groups were between 21 and 40 years. A study conducted in Southwest Ethiopia also reported that malaria was more prevalent among HIV-positive individuals within a similar age range (25–34 years).^31^ Studies in Jos, Nigeria,^4^ Europe,^6^ and Beira, Mozambique^22^ have similarly reported that the prevalence of parasitaemia among HIV patients is usually among this age group. On the other hand, studies conducted on the mainland of Tanzania^32^ and in Nankoma, Uganda^33^ dispute this finding, indicating that the occurrence is typically highest among those under the age of 20, while another in Cross River State, Nigeria discovered that age has no bearing on this.^18^

### Limitations

Abuja is the central area of study, which may not fully represent Nigeria. Notwithstanding, the findings of the study are relevant to the Nigeria situation since the study areas is the capital city of Nigeria where people from different geopolitical regions reside.

### Conclusion

This study reported immunodeficiency as a CD4 count of less than 350 cells/mm^3^. However, this did not have a statistically significant influence on the presence of malaria parasites in the blood of HIV-positive individuals. HIV-positive patients on ART and controls still recorded high rates of malaria. Therefore, these findings suggest that malaria parasitaemia is not an opportunistic infection among HIV-positive patients in Abuja, nor is malaria parasitaemia more common in HIV infection in Nigeria.
